# Tunable Characteristics of Optical Frequency Combs from InGaAs/GaAs Two-Section Mode-Locked Lasers

**DOI:** 10.3390/s24247905

**Published:** 2024-12-11

**Authors:** Dengqun Weng, Yanbo Liang, Zhongliang Qiao, Xiang Li, Jia Xu Brian Sia, Zaijin Li, Lin Li, Hao Chen, Zhibin Zhao, Yi Qu, Guojun Liu, Chongyang Liu, Hong Wang

**Affiliations:** 1 Key Laboratory of Laser Technology and Optoelectronic Functional Materials of Hainan Province, College of Physics and Eletronic Engineering, Hainan Normal University, Haikou 571158, China; 202213085400011@hainnu.edu.cn (D.W.); 201906071316@hainnu.edu.cn (Y.L.); lizaijin@hainnu.edu.cn (Z.L.); lin.li@hainnu.edu.cn (L.L.); 15948713468@163.com (H.C.); 060111@hainnu.edu.cn (Z.Z.); quyi@hainnu.edu.cn (Y.Q.); 068006@hainnu.edu.cn (G.L.); 2 Hainan International Joint Research Center for Semiconductor Lasers, Hainan Normal University, Haikou 571158, China; 3 Shenzhen Pinghu Laboratory, 2 Laolang Road, Shenzhen 518111, China; 4 School of Electrical and Electronic Engineering, Nanyang Technological University, 50 Nanyang Avenue, Singapore 639798, Singapore; jiaxubri001@e.ntu.edu.sg (J.X.B.S.); ewanghong@ntu.edu.sg (H.W.); 5 Temasek Laboratories@NTU (TL@NTU), Nanyang Technological University, Singapore 637553, Singapore; liucy@ntu.edu.sg

**Keywords:** semiconductor lasers, double quantum well lasers, quantum wells, optical frequency comb, mode-locked lasers

## Abstract

We observed tunable characteristics of optical frequency combs (OFCs) generated from InGaAs/GaAs double quantum wells (DQWs) asymmetric waveguide two-section mode-locked lasers (TS-MLLs). This involves an asymmetric waveguide mode-locked semiconductor laser (AWML-SL) operating at a center wavelength of net modal gain of approximately 1.06 µm, which indicates a stable pulse shape, with the power-current(P-I) characteristic curve revealing a small difference between forward and reverse drive currents in the gain region. Under different operating conditions, the laser exhibits the characteristics of OFCs. And the pulse interval in the timing and the peak interval in the frequency domain show a periodic alternating change trend with the increase in the gain current. This tunable characteristic is reported for the first time. The study demonstrates the feasibility of generating tunable optical combs using a monolithic integrated two-section mode-locked semiconductor laser (MI-TS-MLL). This has important reference value for the application of OFCs generated from MI-TS-MLLs or integrated optical chips.

## 1. Introduction

OFCs generated from mode-locked lasers (MLLs) have a wide range of applications, including precision metrology, atomic clock comparisons, timing synchronization, ultra-low-noise microwave generation, calibration of astronomical spectrographs, distance measurement, and laser ranging, as well as direct comb-based spectroscopy [1]. As versatile generators of optical frequencies, frequency combs allow for the precise transfer of phase and frequency information from a highly stable reference across hundreds of thousands of tones in the optical domain.

In recent years, in terms of gain medium, quantum dots (QDs) have enhanced the spectral range and stability of OFCs generated by MLLs due to their broad gain bandwidth and low noise characteristics. However, practical applications still confront challenges such as complex material growth processes and low output power [2]. In contrast, DQW MLLs exhibit outstanding output performance in the near-infrared region due to their mature fabrication processes, excellent gain properties, and strong nonlinear effects. These lasers can achieve OFC outputs with repetition rates reaching GHz or even hundreds of GHz [3]. In terms of chip structure, MLLs are combined using heterogeneous integration technology, which integrates III-V materials with silicon-based photonic platforms, expanding the spectral range of OFCs [4]. This technology faces challenges, such as complex integration processes, interfacial defects, and insufficient output power. For MI-TS-MLLs, a monolithic integration semiconductor laser chip is realized [5]. This integration chip can achieve mode locking and generate a high-repetition-rate and ultrashort pulse laser output [6,7,8]. Therefore, MI-TS-MLL is an important approach for generating OFCs because of its advantages of simplicity, compact size, flexible wavelength tuning, and convenient electrical pumping.

In the past 20 years, MI-TS-MLLs have been continuously improved and optimized. As research progresses on properties such as wide gain bandwidth, ultrafast recovery time, and low bias voltage, they have emerged as efficient, stable, compact, and easy-to-integrate ultrafast laser sources. The performance improvement of MI-TS-MLLs involves four parameters, including wavelength, pulse width, peak power, and repetition rate.

(1) Wavelength

Research on MI-TS-MLLs has gradually shifted towards shorter and longer wavelengths in recent years [9,10,11,12]. In the wavelength range of 1 µm to 1.3 µm, light exhibits high penetrability, allowing it to pass through certain non-metallic materials and biological tissues. Consequently, lasers in this range are widely used in fields such as medical treatment, chemistry, environmental monitoring, and agriculture [13].

(2) Pulse width

The pulse width of MI-TS-MLLs typically ranges from 300 fs to 10 ps [14,15,16,17]. Ultra-short pulses are advantageous for increasing data transmission rates and achieving higher resolution while minimizing damage to biological tissues, making these lasers valuable in optical communications and biomedical applications. Previous research has demonstrated that by using different semiconductor materials (such as GaAs, InGaAsP, GaSb, etc.), along with optimized structural designs for the absorber section and the gain section, the performance of MI-TS-MLLs can be continuously improved, achieving further pulse width compression [18].

(3) Peak power

The peak output power of MI-TS-MLLs typically ranges from 0.2 to 4 W [19,20,21,22]. The peak power primarily depends on parameters such as the lengths of the two sections, the area of the electrical injection region, the quality factor of the optical cavity, and the waveguide loss [23]. By utilizing higher-quality epitaxial materials, standardized manufacturing processes, and optimized structural designs, the gain can be increased while the pulse width is reduced, ultimately producing higher peak output power [24].

(4) Repetition rate

The repetition rate of MI-TS-MLLs generally falls within the range of several GHz to tens of GHz [25,26,27]. This repetition rate is determined by the length and refractive index of the optical cavity [28]. Adjusting the cavity length and structural design are the major methods to tune the repetition rate.

In this work, an InGaAs/GaAs DQW AWML-SL structure was adopted. The structure offers high electroluminescent efficiency and low temperature sensitivity [29], resulting in higher output power. It may achieve higher-order nonlinear effects in the optical waveguide, which are more beneficial for the generation of OFCs. Here, we report on an AWML-SL that emits at approximately 1.06 µm. Its spectral, P-I(power-current), timing, and frequency-domain characteristics have been systematically analyzed. The OFC characteristics and its tunable nature are observed in the chip. These findings provide a promising method for developing on-chip OFCs using MI-TS-MLLs.

## 2. Material Epitaxy and Two-Section Laser Structures

The epitaxial layers of the two-section AWML-SL were grown sequentially on n-type GaAs substrates using metal-organic chemical vapor deposition (MOCVD). From bottom to top, the structure consists of the following: a 200 nm thick n-GaAs buffer layer, a 1300 nm thick n-InGaAsP lower confinement layer with composition and doping concentration gradients (shown in Table 1), an intrinsic 200 nm thick n-In_1−x_Ga_x_As_0.9_P_0.1_ lower waveguide layer, an active region comprising double GaAs/In_x_Ga_1−x_As QW (8 nm GaAs barrier, 7 nm In_0.2_Ga_0.8_As QW), a 200 nm thick low doping p-Al_0.05_Ga_0.95_As upper waveguide layer, and a 900 nm thick p-AlGaAs upper confinement layer with composition and doping concentration gradients (shown in Table 1). Finally, a 250 nm thick p++-type GaAs ohmic contact layer is deposited.

The epitaxial details, including the thickness and doping concentration of each layer, are summarized in Table 1. The design of the epitaxial structure considers the characteristics of current injection and transmission, aiming to minimize waveguide loss in the semiconductor laser. To enhance electrical conductivity and reduce resistivity, asymmetric waveguide structures with varying doping concentrations and thicknesses of AlGaAs and InGaAsP materials, featuring graded composition and doping concentration, are used. Additionally, a double quantum well structure is employed as the active region to increase the excitation and generation efficiency of photons.

**Figure 1:**
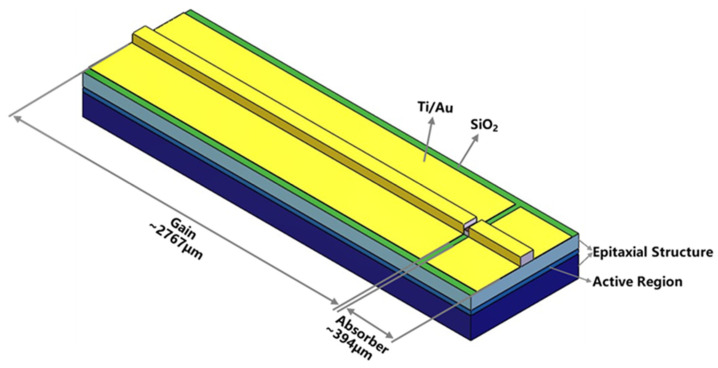
Schematic drawing of the MI-TS-MLL.

Figure 1 shows a schematic drawing of the MI-TS-MLL. The total length of the laser chip is ~3161 µm, consisting of a ~2767 µm long gain section and a ~394 µm long absorber section, separated by a 10 µm wide electrical isolation. The device can achieve mode locking and generate ultrashort pulses by controlling the reverse bias of the absorber and the forward drive current of the gain section. When the current in the gain section and the bias in the absorber section are properly matched, mode-locked pulses with high peak power and a stable optical spectrum can be achieved.

## 3. Process Flow and Experimental Setup

**Figure 2:**
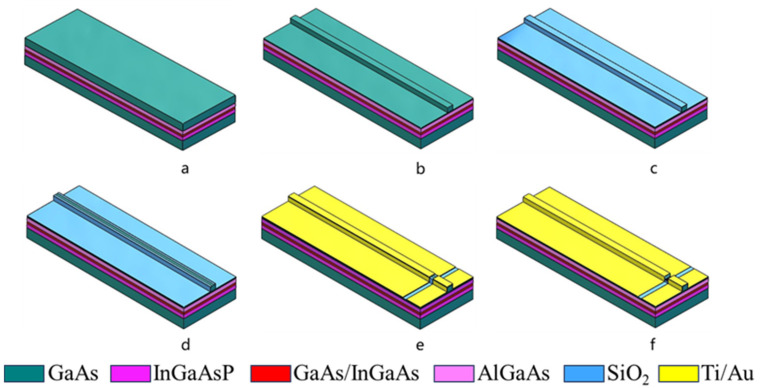
Schematic illustration of the process flow of the MI-TS-MLL (**a**) material epitaxy. (**b**) wet etching. (**c**) 300 nm SiO_2_ insulation layer. (**d**) open electrode window layer. (**e**) P side electrode layers. (**f**) making isolation grooves.

The fabrication process of the MI-TS-MLL is illustrated in Figure 2 and is similar to those described in references [30]. The first photolithography step and the following wet etching process (H_3_PO_4_:H_2_O_2_:H_2_O = 1:1:5) create a ridge-shaped mesa. After removing the photoresist, a 300 nm thick SiO_2_ layer is deposited. The second photolithography step opens a contact window on the ridge, while the exposed SiO_2_ is etched away. The third photolithography step, followed by p-side electrode deposition and lift-off, forms the electrical isolation between the two sections. The resistance is measured to be around ~1.1 kΩ. Next, the substrate is thinned to a thickness of approximately 100 µm, and 5 nm-Ni/25 nm-Ge/100 nm-Au/20 nm-Ni/300 nm-Au are deposited on the n-side. Finally, a rapid annealing process is performed at 410 °C for 30 s in a nitrogen environment. After processing, the sample is cleaved into single chips, and the chips are tested p-side up on a temperature-controlled stage. In addition, we used an optical spectrum analyzer (AQ6370C), a real-time oscilloscope (DSO93004L), and an electrical spectrum analyzer (N9030A) to measure the spectrum, timing, and frequency domain of the laser, respectively. The detailed laser testing method and equipment are described in our previous study [31].

## 4. Experiment Results and Discussion

### 4.1. Laser Gain Analysis

**Figure 3:**
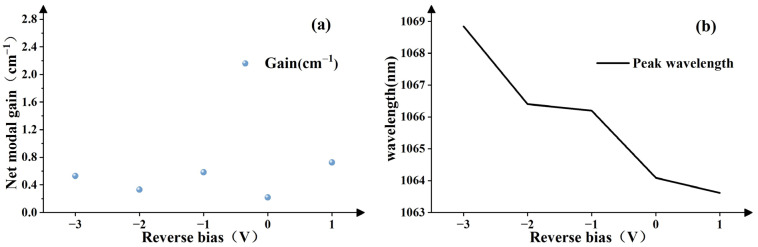
(**a**) Static gain variation with *V_a_*. (**b**) Central wavelength variation with *V_a_*.

The gain was determined through amplified spontaneous emission (ASE) spectroscopy measurements. The net modal gain (*G*_net_) of an MI-TS-MLL can be calculated using the Haki-Paoli method. Specifically, the net modal gain is extracted from the peak-to-valley ratio of subthreshold Fabry-Perot oscillations and is calculated using the following relationship [32]:

(1) $$G_{\mathrm{net}}\left(\lambda \right)=\Gamma g_{m}\left(\lambda \right)-\alpha_{i}=\frac{1}{L}ln\frac{\sqrt{S(\lambda )-1}}{S(\lambda )+1}+\frac{1}{2L}ln(\frac{1}{R_{1}R_{2}})$$

where *Γ* is the confinement factor, *g_m_* is the material gain, and *α_i_* is the internal loss, which is closely related to defects and doping in waveguides and quantum wells (QWs). *S* is the peak-to-valley ratio of the FP resonator, which can be directly obtained from the ASE spectrum. *L* represents the total cavity length (~3.17 mm), and *R*_1_, *R*_2_ are the facet reflectivity (a value of ~0.346 is used for both facets). The ASE spectra were recorded with a wavelength step size of 0.02 nm. Figure 3a shows the net modal gain at the threshold current for five different reverse bias voltage (*V_a_*) conditions. When the *V_a_* is −2 V and 0 V, strong mode locking occurs, causing a significant decrease of the net modal gain. This decrease is significantly different from the change trend of the net modal gain at other *V_a_*. It can be seen that the static gain decreases with the increase of the *V_a_*, which should be caused by the recombination of electrons and holes decreases, leading to decrease in the carrier density concentration and net modal gain. In addition, the diffusion coefficient of electrons and holes also decreases with the increase of the *V_a_*, causing to narrow and decrease the gain. In Figure 3b, it can be seen that the central wavelength gradually red shifts when the *V_a_* increases. This red-shift phenomenon could be explained from the following four aspects: First, the propagation of light pulses in the resonant cavity consumes carriers and causes changes of the refractive index, resulting in that the phase is changed. This effect, known as SPM, not only affects the shape and spectrum of the pulse, but can also cause a red shift in wavelength [33]. Second, explanation from ridge waveguide of MLL: In the gain region, the injected current will increase the carrier density, which leads refractive index of ridge waveguide to increase. As the *V_a_* increases, the recombination between electrons and holes decreases, and the refractive index of the waveguide decreases, causing central wavelength to red-shift. Third, the influence of temperature on red shift cannot be ignored. As *V_a_* increases, the reverse region expands the absorbed photon range and increases photocurrent. Thermalization of carriers is changed will further narrow the band gap and cause emission wavelength to red-shift [34]. Lastly, the energy band (*E_g_*) of the quantum well, built in a polarization electric field, causes the conduction band (*E_c_*) and valence band (*E_v_*) of the quantum well to tilt and reduces the effective width of *E_g_*. This leads to a spatial separation of the electron and hole wave functions, reducing overlap of their wave functions. As a result, the possibility of radiative recombination of electrons and holes is reduced in DQWs, causing luminous efficiency to decrease and luminescence peak to red-shift, which is the Quantum Confined Stark Effect (QCSE) [35,36,37]. Among these factors, the influence of the QCSE dominates.

### 4.2. Laser Spectral Characteristic Analysis

**Figure 4:**
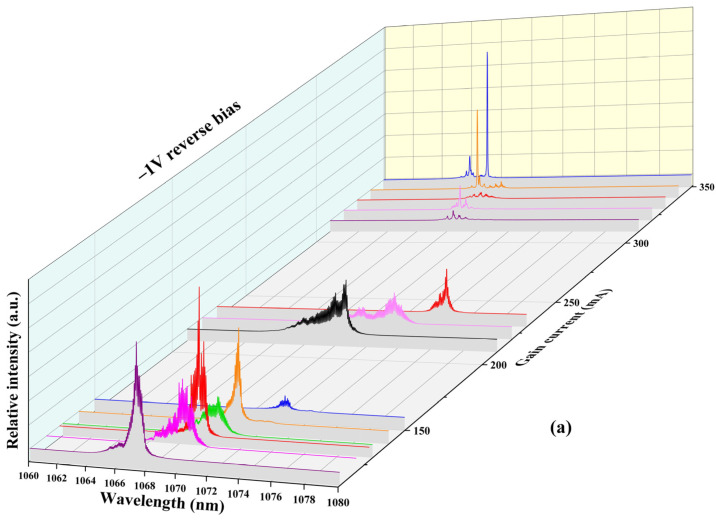

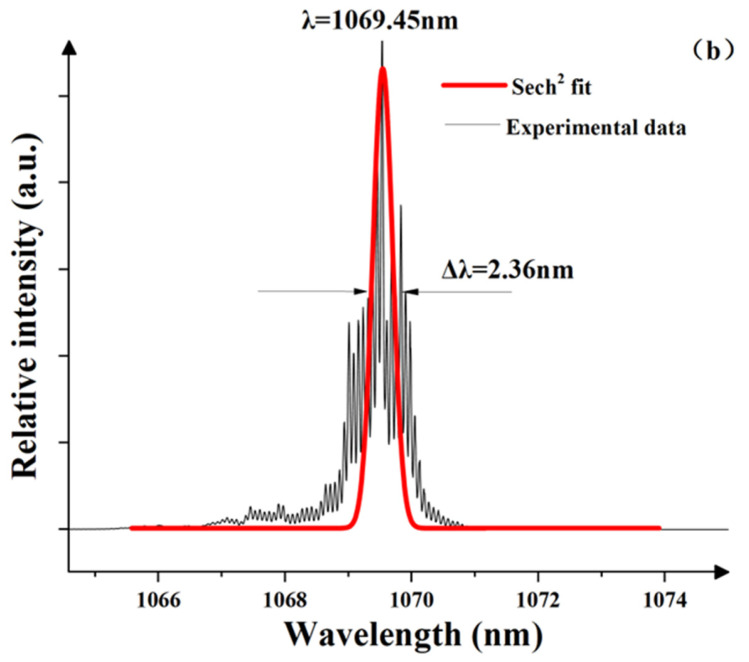
(**a**) Spectra under different *I_g_* with a *V_a_* of −1 V. (**b**) Spectrum (*I_g_* = 128 mA).

Figure 4a shows the evolution of the laser spectra as the gain current (*I_g_*) increases (*V_a_* is fixed at −1 V). When the *I_g_* is 128 mA, fitting with the sech² function is performed, as shown in Figure 4b. The central wavelength and full width at half maximum (FWHM) of the AWML-SLs are found to be 1069.45 nm and 2.36 nm, respectively. The data were fitted using the sech^2^ function, resulting in a fitting curve that closely aligns with the distribution of the data curve. Therefore, we guess that the pulse peak distribution may be a soliton state. The AWML-SL laser demonstrates stability at a wavelength of ~1.06 µm.

### 4.3. PI Characteristic Analysis

**Figure 5:**
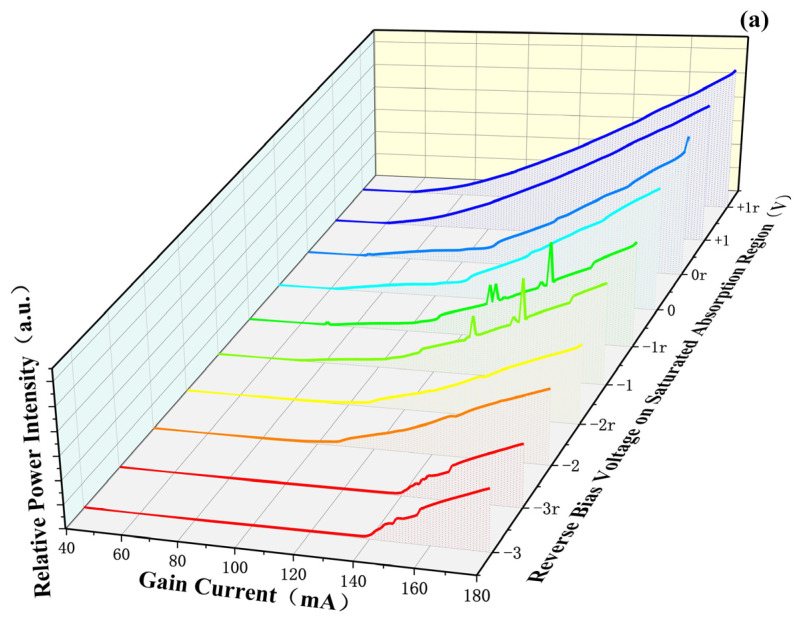

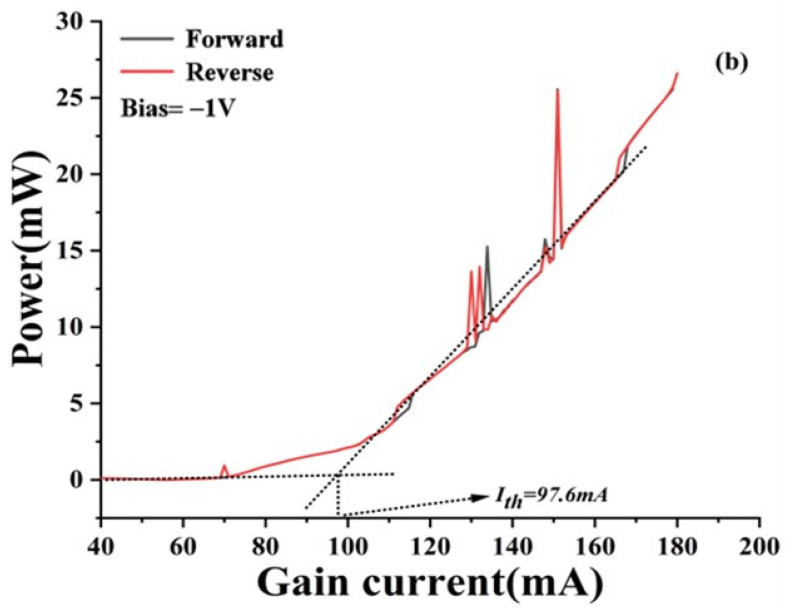
(**a**) P-I curve (r indicates that the drive current of the gain section is changed from high to low). (**b**) P-I curves are comprised at −1 V (−1r) bias (part typical hysteresis characteristic).

Figure 5a presents the test results for the power-current (P-I) characteristics of the ~1.06 µm MI-TS-MLL under various absorber reverse biases. It can be observed that as the *V_a_* increases, the threshold current increases, while the output power decreases and the slope efficiency decreases. These changes are caused by the larger absorption induced by the absorber.

When the *V_a_* is set to −1 V, the MI-TS-MLL exhibits Q-switching (glitch phenomenon), which is a mode of MLL operation also known as “Q switching” or “giant pulse.” In this mode, the photon storage within the resonator is periodically increased and then rapidly released, resulting in ultrashort, high-peak-power laser pulses. This demonstrates that *V_a_* can effectively control the operating state. In order to study the Q-switch phenomenon, the P-I curve is further analyzed when the *V_a_* is −1 V under reverse and forward current drive, respectively, as shown in Figure 5b. In the forward current drive process, the carrier density is increased, which leads to heat accumulation. In addition, due to the interaction between carriers and photons, this leads to an increase in the relative refractive index, which results in a relatively enhanced ability of the waveguide to confine photons. In the reverse current drive process, the carrier density in the active region is reduced. The refractive index is relatively reduced, leading to a relatively weakened ability of the waveguide to confine photons. Therefore, it is easier to achieve lasing for the forward than the reverse. According to temperature influence, the reverse current drive can effectively reduce the thermal effect of the chip. Considering comprehensively, the different density of exciting photons between forward and reverse drive is not obvious, and the peak power of the forward drive is slightly higher than that of the reverse drive.

### 4.4. Time-Frequency Analysis

**Figure 6:**
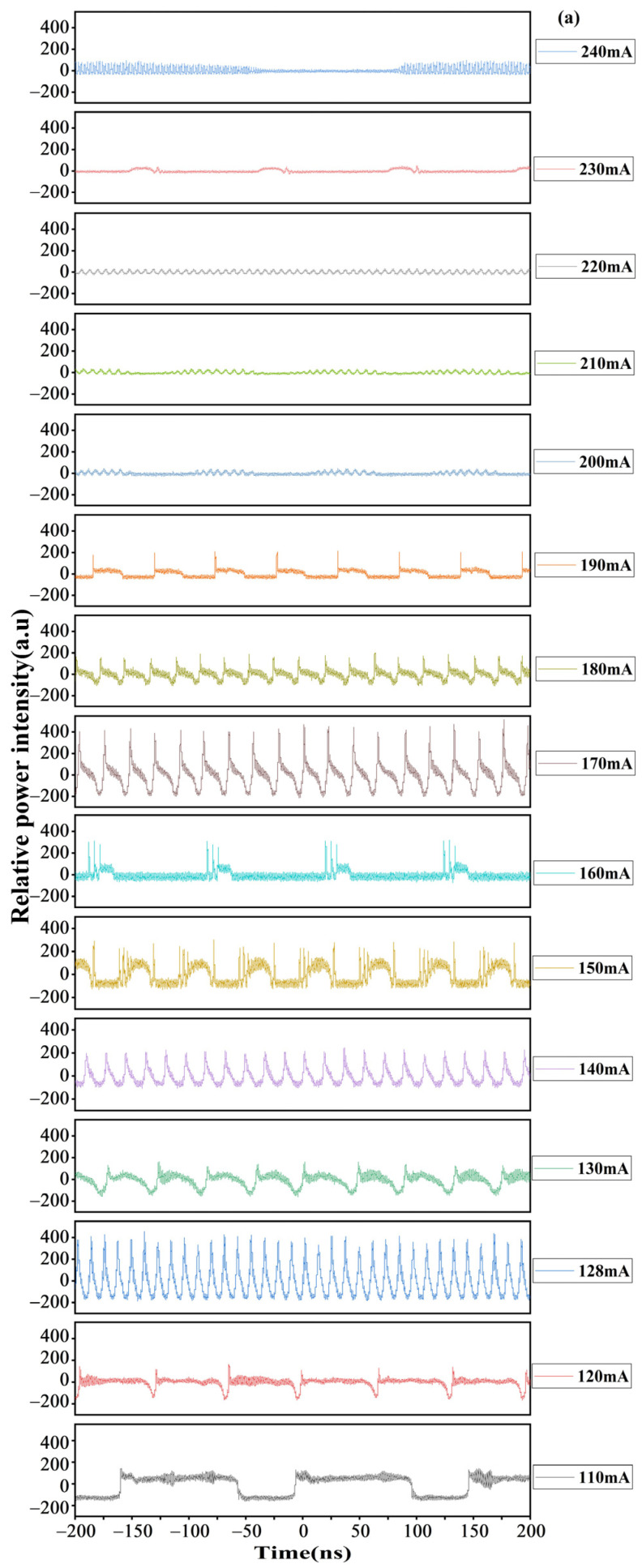

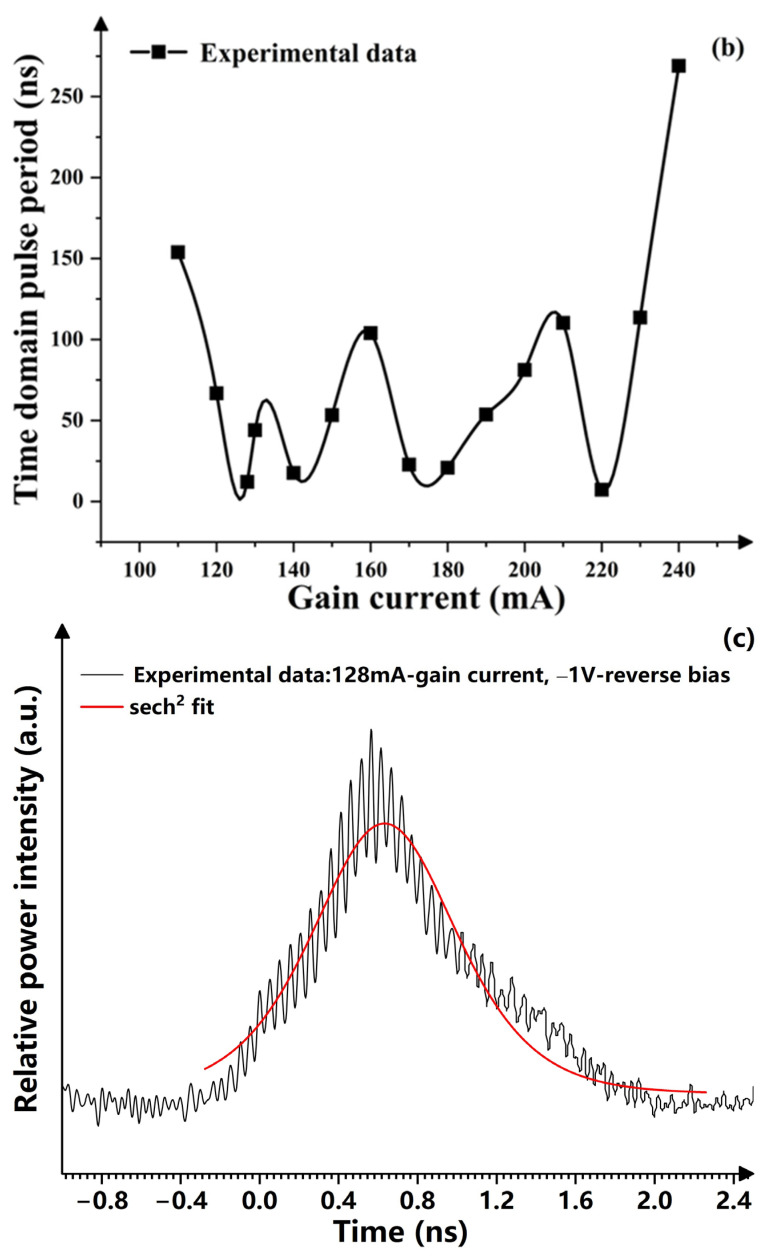
(**a**) The timing diagram of ~1.06 µm AWML-SL under different injection current conditions (the *I_g_* is from 110 to 240 mA, with a 10 mA steps, the *V_a_* is −1 V). (**b**) The timing pulse period varies with *I_g_*. (**c**) The generated pulse shape (the red line is the timing sech^2^ fitting curve).

The test temperature is set at 25 °C, and the timing characteristics are tested in the current range from 110 mA to 240 mA. The timing characteristic curves under different *I_g_* conditions are shown in Figure 6a. At 128, 140, 170, 180, and 190 mA, the modes have been locked, forming various timing combs. Due to longitudinal modes competition, thermal effects, and gain current noise, each locked mode is independent and forms its own pulse fluctuations in the timing. In addition, the pulse period shows a periodic alternating trend of increasing and decreasing with *I_g_*, as shown in Figure 6b. When the *I_g_* increases within a certain range, the gain gradually increases, and the interaction between modes enhances, resulting in a shortened signal period and the formation of high peak and narrow pulse width pulses. However, as the *I_g_* increases further, thermal effects and longitudinal mode competition problems become obvious. These effects are accompanied by mode locking, increasing the period of the pulse and forming low peak power and wide pulses. Therefore, the *I_g_* has a modulating effect on the pulse period. As the *I_g_* increases, the pulse produces a periodic trend of alternating increases and decreases.

When the *I_g_* is 128 mA, the period of the timing signal is 12.1 ps, the pulse is stable, and there is no obvious multi-mode or strong noise. The hyperbolic secant sech^2^ function is fitted, and the corresponding chirp-free optical pulse time-bandwidth product is 0.315.

Through the basic formula of the Time-Bandwidth Product (TBP):

(2) TBP=τ•B

*τ* represents the pulse width, the unit is second (s); *B* represents the spectral bandwidth, the unit is Hertz (Hz); Formula (2) is the ideal value under a chirp-free condition.

(3) $$B=\frac{\Delta \lambda }{{\lambda_{0}}^{2}}c$$

where Δ*λ* is the peak width at half maximum of the spectrum, *λ*_0_ is the center wavelength of the laser, and *c* is the speed of light. Here, Δ*λ* = 2.36 nm, *λ*_0_ = 1069.45 nm, *c* = 3 × 10^8^ m/s. From Equation (3), the spectral bandwidth *B* is 6.19 × 10^11^ Hz, and according to Equation (2), the pulse width of ~1.06 µm AWML-SL is 0.51 ps.

According to the formula:

(4) $$P_{peak}=\frac{P_{avg}}{\tau f}$$

where *P_peak_* represents the peak power, *P_avg_* is the average output power, *τ* is the pulse width, and *f* is the repetition rate. Watt and several watts level peak output power can be obtained under chirp-free.

Figure 6c shows the timing sech^2^ function fitting under 128 mA *I_g_*. According to the fitting results, the laser can generate narrow pulse widths and sub-picosecond pulses. In addition, the fitting curve and the experimental data show consistent agreement, indicating that the laser can stably generate picosecond and sub-picosecond pulses.

**Figure 7:**
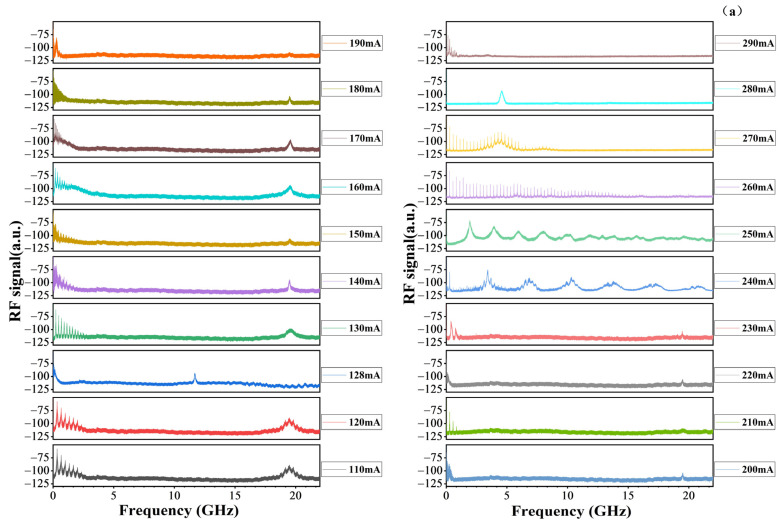

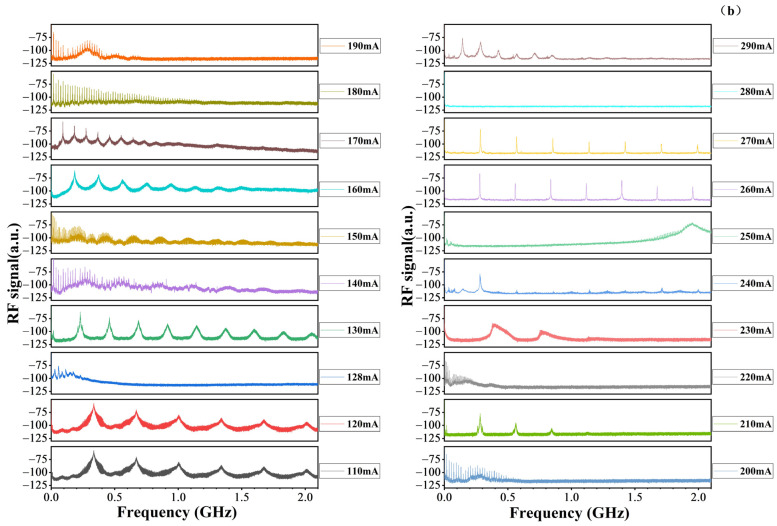
(**a**) The frequency domain diagram of ~1.06 µm AWML-SL under different injection current conditions (The current is from 110 to 300 mA by a 10 mA steps, and the *V_a_* is −1 V on the absorber region). (**b**) The partial frequency domain diagram (The current is from 110 to 300 mA by a 10 mA steps, and the *V_a_* is −1 V on the absorber region).

Figure 7a,b show the frequency domain diagram of a ~1.06 µm AWML-SL. They demonstrate that the MI-TS-MLL exhibits multiple frequency peaks with uniform frequency distribution under specific operating conditions. The frequency spectrum line spacing shows a periodicity of alternating increases and decreases with the increasing current.

As we all know, in a laser resonator cavity, the condition forming stable standing waves is [38]:

(5) $$L=\frac{m\lambda }{2n}$$

By using the wavelength formula *λ* = *c*/*f*, the frequency can be obtained as:

(6) $$f=\frac{mc}{2nL}$$

where *n* is the refractive index of the laser resonator cavity, *m* is a positive integer, and *L* is the length of the resonant cavity. In addition, the relationship between the emission wavelength and the energy band is *λ* = 1.24/*E_g_* [39]. Thus, the relationship between frequency and the energy band can be derived as:

(7) $$f=\frac{cE_{g}}{1.24}$$

As the *I_g_* increases, the internal core temperature of the MI-TS-MLLs rises. This leads to an increase in the refractive index, and due to the thermal expansion of the semiconductor material, the cavity length increases. Moreover, it results in a narrowing of the energy band (*E_g_*). According to Formulas (6) and (7), this will lead to a decrease in frequency. Furthermore, an increase in *I_g_* results in the MI-TS-MLLs exhibiting dynamic changes in the number of longitudinal modes and affecting the mode-locking state of the MI-TS-MLLs. If the value of *m* becomes larger, the frequency will increase rapidly; on the contrary, the frequency will decrease. These factors lead to a periodic trend in which the spectral line spacing alternates between increases and decreases. Figure 7b shows that the spacing between the frequency peaks is equal, and there is a significant difference in peak intensity. By changing the *I_g_*, the pulse peak period modulation can be realized. However, its controllability still needs to be further studied. When the *V_a_* in the absorber region is −1 V, and the *I_g_* is 140 mA, 150 mA, 180 mA, 190 mA, and 200 mA, respectively, a definite OFC phenomenon is observed. OFCs are mainly generated in the low-frequency region and are affected by Kerr nonlinear effects [40].

## 5. Conclusions

A monolithic integrated InGaAs/GaAs TS-MLLs emitting at ~1.06 µm is demonstrated. This study obtained the operating characteristics under different conditions by testing and analyzing the P-I curve, spectrum, timing, and frequency domain characteristics. When the *I_g_* is 128 mA, the laser output center wavelength and FWHM are ~1069.45 nm and 2.36 nm, respectively. The output pulse width is calculated to be ~0.51 ps under chirp-free conditions. The peak output power is calculated to be up to one watt or several watts while the chip is operating under a chirp-free condition. It is an ideal result, but further testing is needed. The tunable characteristic of the OFC is clearly observed for the chip. The *I_g_* has a modulation effect on the timing pulse period and the frequency domain peak period. However, the precise controllability of this tunable characteristic needs to be further studied. Additionally, OFCs are mainly generated in the low-frequency region and are affected by nonlinear effects. These discoveries provide important reference for generating tunable OFCs from MI-TS-MLLs. Especially in practical applications, the tunable characteristics of the OFCs become crucial when OFCs are used as light sources for TS-MLLs generation.

## Figures and Tables

**Table 1:** Epitaxial structures and materials.

Material	Thickness (nm)	Doping Concentration (cm^−3^)
GaAs	250	P, >2 × 10^19^
Al_0.55_Ga_0.45_As	300	P, >8 × 10^17^
Al_0.35_Ga_0.65_As	300	P, >8 × 10^17^
Al_0.3_Ga_0.7_As	300	P, >8 × 10^17^
Al_0.05_Ga_0.95_As	200	P, >8 × 10^16^
GaAs	8	barrier
In_0.2_Ga_0.8_As	7	quantum well
GaAs	8	barrier
In_0.2_Ga_0.8_As	7	quantum well
GaAs	8	barrier
In_0.04_Ga_0.96_As_0.9_P_0.1_	200	non-doped
In_0.14_Ga_0.86_As_0.7_P_0.3_	300	n, 8 × 10^16^
In_0.24_Ga_0.76_As_0.5_P_0.5_	100	n, 8 × 10^17^
In_0.33_Ga_0.67_As_0.3_P_0.7_	100	n, 8 × 10^17^
In_0.42_Ga_0.58_As_0.1_P_0.9_	800	n, 8 × 10^17^
GaAs	200	n, 2 × 10^18^

## Data Availability

Data underlying the results presented in this paper are not publicly available at this time but may be obtained from the authors upon reasonable request.
